# Data-driven approaches used for compound library design, hit triage and bioactivity modeling in high-throughput screening

**DOI:** 10.1093/bib/bbw105

**Published:** 2016-10-27

**Authors:** Shardul Paricharak, Oscar Méndez-Lucio, Aakash Chavan Ravindranath, Andreas Bender, Adriaan P IJzerman, Gerard J P van Westen

**Affiliations:** 1 Centre for Molecular Informatics, Department of Chemistry, University of Cambridge, Lensfield Road, Cambridge, United Kingdom; 2 Division of Medicinal Chemistry, Leiden Academic Centre for Drug Research, Leiden University, RA Leiden, The Netherlands; 3 Facultad de Química, Departamento de Farmacia, Universidad Nacional Autónoma de México, Avenida Universidad 3000, Mexico City, Mexico

**Keywords:** phenotypic assays, library design, screening paradigms

## Abstract

High-throughput screening (HTS) campaigns are routinely performed in pharmaceutical companies to explore activity profiles of chemical libraries for the identification of promising candidates for further investigation. With the aim of improving hit rates in these campaigns, data-driven approaches have been used to design relevant compound screening collections, enable effective hit triage and perform activity modeling for compound prioritization. Remarkable progress has been made in the activity modeling area since the recent introduction of large-scale bioactivity-based compound similarity metrics. This is evidenced by increased hit rates in iterative screening strategies and novel insights into compound mode of action obtained through activity modeling. Here, we provide an overview of the developments in data-driven approaches, elaborate on novel activity modeling techniques and screening paradigms explored and outline their significance in HTS.

## Introduction

Traditionally, knowledge from the areas of pharmacology and medicinal chemistry is combined to design potentially active compounds for testing [1–3]. However, improvements in robotics, automation and combinatorial chemistry led to the development and increasing use of high-throughput screening (HTS). HTS allowed rapid screening of large compound libraries [3–6] and enabled pharmaceutical companies to explore the bioactivity profiles of compounds covering a larger amount of chemical space [7] with the intention to increase the chances of identifying (diverse) hits for further investigation.

However, multiple nontrivial challenges still exist in HTS. First, the effectiveness in HTS directly depends on the compounds screened, and therefore the design of compound libraries is of great importance [8]. Second, HTS at times cannot be performed for certain assays (such as those involving complex biological systems that do not allow for mass production), making it an unviable option in such cases [3, 9]. Third, measurement errors and artifacts related to both assay miniaturization and screening technologies used can complicate the analysis of screening results, making effective triage for follow-up screens a prerequisite for successful campaigns [8]. Finally, despite improvements in screening technology, HTS campaigns are still costly because of the large amount of resources required in relation to the number of active compounds discovered [6]. Moreover, Macarron *et al.* [10] describe that much of the cost associated with HTS is because of the upfront investments in HTS infrastructure and assay development, and that the cost per campaign is estimated to be 10–20% higher relative to other methods.

The above-mentioned drawbacks highlight the need for intelligent measures to increase efficiency in HTS. This need, fueled by the increasing amount of bioactivity data available [11] and advances in cheminformatics, has prompted numerous data-driven and computational efforts to improve various aspects of HTS [12–15].

Approaches suggested for library design include focused design for target classes such as G Protein-Coupled Receptors (GPCRs) or kinases with many known active chemotypes [2, 16, 17], and diversity-based design for target classes with few known active chemotypes or for phenotypic assays. For the latter, structural diversity in screening libraries is preferred, as this can increase the chances of finding multiple promising scaffolds for further development across a wide range of assays [18, 19]. In addition, much effort has been made to improve hit triage [20–24], as the selection of actives from primary screens for follow-up screening is not trivial because of the low signal-to-noise ratio in HTS. Finally, virtual HTS (vHTS) approaches are used to prioritize compounds for testing, based on computational model predictions. Recently, ample progress has been made in this area, which we will discuss in detail below [23, 25–31].

In this review, we summarize the recent developments in data-driven applications to improve effectiveness in HTS and discuss the strengths and limitations of these methods. We briefly discuss library design, experimental error management and hit triage. Furthermore, we elaborate on recent developments in bioactivity modeling. Finally, we explore some recently introduced new screening paradigms and highlight their use in further improving efficiency.

## Diversity-based library design for targets with few known active chemotypes or phenotypic assays

**Figure 1:**
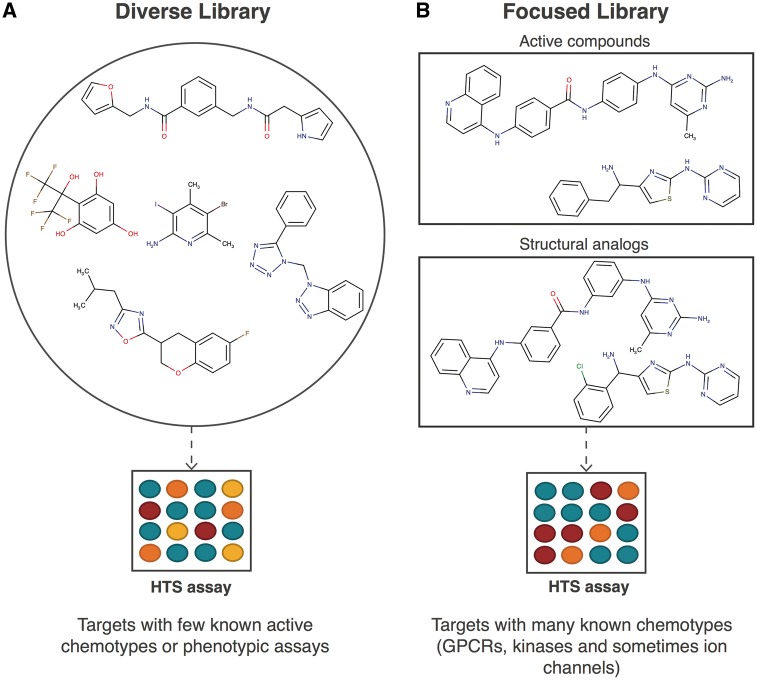
Diverse libraries compared with focused libraries. Structurally diverse libraries are used to efficiently explore relevant chemical space for targets with few known active chemotypes or for phenotypic assays [34] (**A**). This is performed to provide multiple starting points for further development. Example structures were taken from the ZINC lead compounds library [35], and PAINS [36] were omitted. Owing to the diversity of the compounds tested, a wide range of activities can be observed: from inactive (blue) through somewhat active (yellow) and moderately active (orange) to highly active (red). By contrast, focused libraries are often designed for targets with many known active chemotypes, such as GPCRs, kinases and, in some cases, ion channels (**B**). Here, example structures were taken from Harris *et al.* [37] and Fernández-de Gortari and Medina-Franco [38], and PAINS [36] were omitted. These libraries focus around active chemotypes found previously, for instance, through diversity-based screening [2, 37, 39, 40]. Here, analogs often exhibit fewer differences in activity, as the presence of many more similar compounds will more likely result in multiple actives compared with diverse libraries.

While over 10^63^ drug-like molecules possibly exist [32], likely only a fraction of these molecules is therapeutically relevant [33]. Therefore, efficient exploration of relevant chemical space is important for targets with few known active chemotypes or phenotypic assays [34]. Diversity-based library design addresses this need by optimizing biological relevance and compound diversity to provide multiple starting points for further development (Figure 1A) [18, 19]. However, diversity is an ambiguous term [41, 42], as it can be based on a wide range of chemical descriptors (fingerprint-based [43], shape-based [44, 45] or pharmacophore-based [46]) or even biological descriptors (affinity fingerprints [27, 29, 47] or high-throughput screening fingerprint, HTS-FP [25]), potentially yielding contrasting results [48]. Chemical descriptors characterize compounds in terms of structural and/or physicochemical properties. A comprehensive study over 115 HTS assays by Martin *et al.* [49] showed that while structural similarity correlates with similarity in bioactivity, the chance that a compound similar to an active compound (Tanimoto similarity ≥0.85 based on Daylight fingerprints [50]) is itself active is only 30%. By contrast, biological descriptors represent compound phenotypic effects and bioactivity against the druggable proteome. Recent studies at Novartis have shown that these biological descriptors often significantly outperform chemical descriptors regarding hit rate and scaffold diversity in HTS campaigns, and can even be used in conjunction with chemical descriptors for augmented performance [14, 24, 25]. While biological descriptors have been used for selecting compounds from an existing library with great success, they cannot directly be used for design and purchase of new compounds that lack biological data.

## Focused library design for targets with many known active chemotypes

Contrary to diversity-based libraries designed for targets with few known active chemotypes, focused screening libraries are often designed for well-studied targets, such as GPCRs, kinases and, in some cases, ion channels. Focused libraries center around active chemotypes found through diversity-based screening (Figure 1B) [2, 37, 39, 40] and can be selected from larger diversity-based libraries using structure-based and/or ligand-centric similarity metrics as shown by Tan *et al.* [51]. The knowledge of binding mode (such as hinge binding, DFG-out binding and invariant lysine binding for kinases) is often used during library design to develop ligands with desirable properties [37]. Overall, for target classes with known active chemotypes or with additional information on structure–ligand interaction, focused libraries lead to higher hit rates than diversity-based libraries. This was evidenced in the study by Harris *et al.* [37] where 89% (kinase-focused) and 65% (ion channel-focused) of focused libraries led to an improved hit rate compared with their diversity-based counterparts. However, despite higher hit rates, focused approaches may not effectively sample diverse chemical space. This could be problematic when certain chemotypes are to be avoided because of off-target effects or intellectual property reasons. Hence, focused libraries are not necessarily a replacement for diversity-based approaches, even for well-studied target classes. Harper *et al.* [52] described a quantitative method to design a suitable library taking into account both compound diversity and the inclusion of known active chemotypes. A deeper discussion of the design of chemical libraries can be found in the following book chapter [53].

## Management of experimental error in HTS

**Figure 2:**
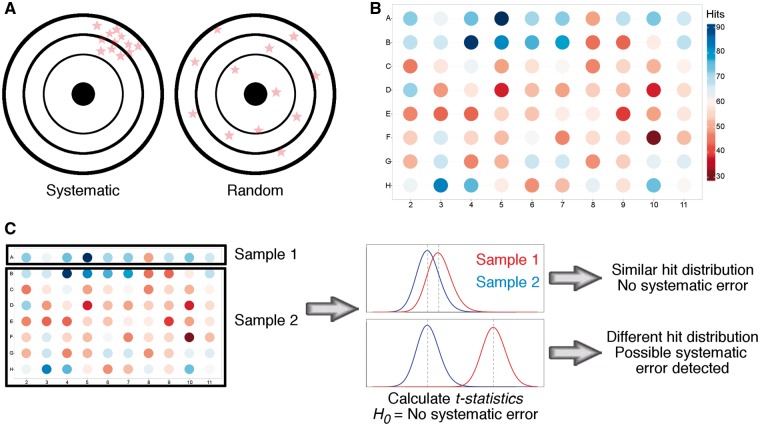
Graphical representation of the differences between systematic and random errors. Systematic errors are associated with consistent over- or underestimated activity across the screening collection. By contrast, while random errors are usually caused by noise and have a low impact on the overall results, they do not present any pattern, which makes their identification more difficult (**A**). We show an example of systematic error in the McMaster University experimental HTS assay [57] (**B**). Here, the number of hits in each well across 1250 plates is shown. In general, wells located in rows A and B presented a higher hit rate than those at the center of the plates, exemplifying how the well position can be associated with a systematic error. Systematic errors can be detected using the Student’s *t*-test [56], for example (**C**). Here, measurements from one row or column (Sample 1) are compared with those of the remainder of the plate (Sample 2). When mean hit values of Sample 1 are significantly different from mean values of Sample 2, a systematic error is detected.

As any experimental technique, HTS is not exempt of experimental errors, and the large amount of data obtained from these campaigns make their detection challenging [54, 55]. In general, errors in HTS can be classified as random or systematic. Random errors are usually caused by noise and have a low impact on the overall results, as no methodical bias is introduced. By contrast, systematic errors are associated with consistent over- or underestimated activity across the screening collection [56, 57] (Figure 2). Many procedural, technical and environmental reasons exist for systematic errors, such as malfunctioning robots, readout interpretation from plates, reagent evaporation, degradation of target protein or cell decay [56, 58]. Awareness of these problems has prompted efforts to find new ways of detecting and correcting these errors to achieve a better selection of compounds.

Statistics plays an important role in the analysis and detection of errors in HTS [55, 59]. Dragiev *et al.* [56] described the use of three statistical approaches to detect systematic errors in HTS data: the Student’s *t*-test, the *χ*^2^ goodness-of-fit and the discrete Fourier transform (DFT) in conjunction with the Kolmogorov–Smirnov test. More specifically, the Student’s *t*-test can be used to find systematic errors in both hit distribution surfaces (i.e. counts of hits in each particular well of the plate) or across independent plates. As shown in Figure 2C, this test compares the hit distribution of each row or column with the rest of the plate. If the hit distribution of each row is similar to the rest of the plate based on *t* statistics, *H*_0_ is true and there is no systematic error. By contrast, if the hit distributions are different (*H*_0_ is false), a systematic error is detected.

The *χ*^2^ goodness-of-fit follows a similar procedure to that of the Student’s *t*-test, but it can be only applied when using hit distribution surfaces. The *χ*^2^ goodness-of-fit ensures that the number of hits in each well is not significantly different from an expected value, which is the total number of hits across the entire surface divided by the number of wells. The third method entails the use of DFT to detect frequencies of signals that repeat every fixed number of wells to generate a density spectrum. Subsequently, a null density spectrum corresponding to randomly distributed hits across the plate is generated. Finally, the DFT density spectrum is compared with the null density spectrum using the Kolmogorov–Smirnov test to determine the existence of systematic errors. Together, all these methods can be used to measure the error in the hit distribution surface, to measure errors for samples with different sizes and to analyze signal frequency. In a more recent study, Dragiev *et al.* [58] proposed two widely used methods, namely Matrix Error Amendment and partial mean polish, for correcting errors in HTS with improved results. A deeper discussion of statistical methods for normalization and error correction can be found in two informative reviews [55, 60].

A wide range of software packages [61–65] is available to facilitate analysis and error correction of HTS data (Table 1). Earlier programs such as HTS-Corrector [61] enable the analysis of background signals, data normalization and clustering. Building on this foundation, more recent and advanced software such as HTS navigator [64] provides features such as loading multiple data sets, visualization and cheminformatics analysis. The key benefit is that the user can perform a larger part of the analysis on a single platform.

**Table 1:** An overview of software available for HTS data analysis

Software name	Description	Reference (year)
HTS-Corrector	Analysis and error correction of HTS data	[61] (2006)
HDAT	Web-based HTS data analysis	[62] (2013)
HCS-Analyzer	Analysis and error correction of high-content screening data	[63] (2012)
HTS navigator	Cheminformatics analysis, visualization and error correction of HTS data	[64] (2014)
WebFlow	Analysis of HTS cytometry data	[65] (2009)

*Note* Most software packages enable data analysis and error correction, and more advanced software such as HTS navigator allows for both cheminformatics analysis and visualization.

## The importance of hit triage

The goal of HTS triage is to prioritize a subset of the large number of detected actives in the primary screen for further investigation and optimization [8]. However, the analysis of HTS data can be complicated by large library sizes and experimental errors caused by artifacts related to assay miniaturization or screening technologies used. A number of filters such as rapid elimination of swill, pan-assay interference compounds (PAINS), the rule of three and the rule of five are routinely used to discard compounds with undesirable properties (e.g. promiscuity, poor physicochemical properties or presence of problematic functional groups) [8, 66–69]. While ideally this should take place at the library design stage, analysis of historical HTS data requires that this filtering be applied at the triage stage as well, as often historical assays contain undesirable compounds because of improper filtering at the time of design. This is followed by the selection of diverse sets of actives for follow-up testing based on potency and scaffold structure–activity relationships (SAR) [8, 69, 70].

Chemically diverse compound sets are preferred over sets comprising many analogs, as the former allows multiple starting points for compound optimization, increasing the overall chances of success. Nevertheless, some analogs in the screening set are desired to enable SAR analysis. Nilakantan *et al.* [71] and Lipkin *et al.* [72] suggested a middle-of-the-road approach by designing diverse libraries with at least 50 or 200 analogs per scaffold, respectively, with the intention of reducing the chances of missing an active scaffold series while still covering a significant amount of chemical space. HTS data are used to develop models for each chemical class (i.e. scaffold), and active classes are identified based on the relative prevalence of (primary) hits within the class. Actives belonging to an active class are prioritized over those belonging to poorly performing classes, as the latter may more likely be false positives. Additionally, rescuing false negatives is also important; a number of data mining approaches have been explored to this end [73]. Often, SAR analysis takes place after secondary screens, and concentration–response curves have been performed on a much smaller set of selected compounds. However, a study by Varin *et al.* [70] demonstrated the benefit of including this analysis immediately after the primary HTS screen. Here, primary screening data were preferred over secondary data because of its size and completeness, despite the lower quality. Hit triage results can be organized in a scaffold tree with well-defined chemical entities, allowing for intuitive classification and decision-making from a medicinal chemist’s point of view [74].

## Developments in virtual HTS and new screening paradigms

vHTS is used in parallel to intelligent library design, error management and hit triage. vHTS attempts to learn from existing biochemical or phenotypic data and prioritizes subsets of much larger screening libraries for experimental testing.

The wide range of techniques used in vHTS can mainly be divided into two groups: structure-based and ligand-centric vHTS. The former relies on three-dimensional structural information (X-ray crystal or NMR structure) of the target protein to study possible interactions with compounds in the screening library [75, 76]. The most common structure-based method is molecular docking, which predicts a binding pose for the compound and assigns a score based on the interactions formed in the protein–ligand complex, representing the suitability for experimental testing. By contrast, ligand-centric approaches exploit structural information of known active compounds to identify new actives. A number of ligand-centric approaches exist: pharmacophore modeling [77, 78], quantitative structure–activity relationship modeling [79] and similarity searching [80] among others [75, 76].

The low cost and resources required for vHTS combined with the introduction of large public bioactivity databases [11] facilitate its application to many drug discovery campaigns. This has resulted in numerous success stories: the discovery of inhibitors/ligands of DNA methyltransferases (DNMTs) [81, 82], kinases [83, 84], GPCRs [85, 86] and other relevant targets (Table 2) [87, 88]. Nevertheless, the success of vHTS depends on initial data quality and validation procedures.

**Table 2:** Successful applications of vHTS

Target	Main contribution	Method	Reference (year)
DNMT	Olsalazine, an anti-inflammatory drug as DNMT inhibitor	Ligand-centric	[81] (2014)
DNMT	Nanaomycin as selective DNMT3b inhibitor	Structure-based	[82] (2010)
Chk-1 kinase	Thirty-six inhibitors with IC_50_ values between 68 nM and 110 μM	Ligand-centric, pharmacophore-based and structure-based	[83] (2003)
JAK3	Identification of a diazaindazole scaffold (IC_50_ = 98 nM)	Ligand-centric and structure-based	[84] (2011)
NPY5 receptor	Eleven antagonists (IC_50_ ≤ 1 μM)	Ligand-centric and pharmacophore-based	[85] (2005)
Adenosine receptors	Six high-affinity adenosine receptor ligands	Ligand-centric and binding pocket-based	[86] (2012)
Neurokinin-1 receptor	One compound with IC_50_ = 0.25 μM	Pharmacophore-based and structure-based	[87] (2004)
mGlu4 receptor	Six agonists from a library of 720 000 compounds	Structure-based	[88] (2005)

*Note* Additional examples have been reviewed by Matter and Sotriffer [89].

**Figure 3:**
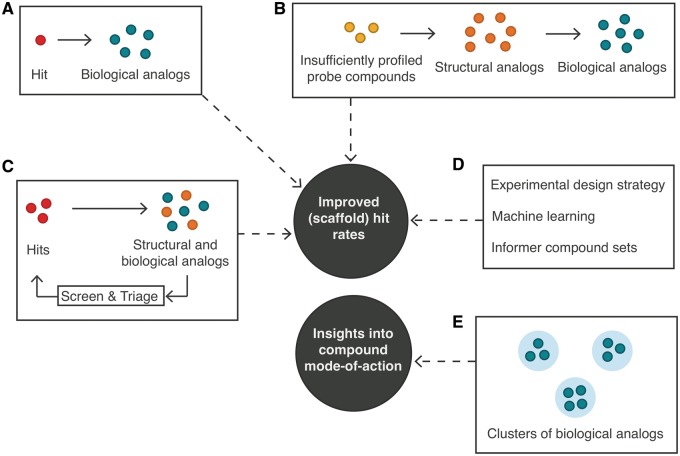
Overview of recent studies improving (scaffold) hit rates and providing insights into compound mode of action. Describing compound bioactivity across ∼200 assays at Novartis, Petrone *et al.* [25] took the concept of bioactivity-based similarity to an unparalleled level. Here, biological analogs of hits were prioritized for testing (**A**). Later studies leveraged bioactivity profiles of structural analogs of poorly characterized compounds to select subsets of compounds for virtual screening [24] (**B**), or used a screening strategy using biological and chemical similarity metrics in parallel to iteratively expand around hits from multiple rounds of screening [3] (**C**). Further improvements resulted from changes in experimental design strategy [93], machine learning methods for predicting actives [23] and informer sets for routine exploratory screening [94] (**D**). Other studies used bioactivity-based similarity searching for mode-of-action analyses at Novartis [91], Roche [92] and in the public domain [26] (**E**).

With the recent advent of the ‘HTS-FP’, which describes compound bioactivity across ∼200 biochemical and cell-based assays at Novartis [25], the concept of bioactivity-based similarity was taken to an unparalleled level. HTS-FP builds on the idea of affinity fingerprints [27, 29, 90], allowing a bioactivity-based comparison of compounds. Petrone *et al.* [25] demonstrated the benefit of this descriptor over state-of-the-art chemical descriptors in vHTS and scaffold hopping. This study formed the basis for a body of work on using bioactivity-based similarity searching for mode-of-action analyses [24, 26, 91, 92] and bioactivity modeling, resulting in enhanced (scaffold) hit rates [3, 23, 24, 93] (Figure 3). Building on this success, a public version of HTS-FP was later designed based on PubChem bioactivity data [95].

Wassermann *et al.* [24] developed a method named ‘bioturbo similarity searching’. For insufficiently profiled probe compounds, bioactivity profiles of structural analogs were leveraged to select subsets of compounds for virtual screening. Screening these subsets led to higher (scaffold) hit rates compared with when only structural similarity metrics for expansion around probe compounds were used. Further work addressed the use of bioactivity-based similarity searching for target prediction [26, 91], detection of frequent hitters [26, 69] and iterative selection of activity-enriched subsets of the compound collection for screening [3]. Driven by the gained momentum in machine learning [96], a comprehensive benchmarking of machine learning classifiers in conjunction with chemical and biological descriptors was performed, with the overall net result that fusing both HTS-FP and chemical descriptors led to the best performance [23]. Moreover, a study by Paricharak *et al.* [94] described the implementation of an active learning approach to derive ‘informer compound sets’ <10% of the entire screening collection. Such sets were shown to provide improved predictivity over the remainder of the screening collection compared with the randomly selected training sets. Hence, the availability of these sets enables routine exploratory screening in an assay-agnostic manner for improved hit expansion [94]. The concept of bioactivity-based similarity has also been inspected from the (cellular or protein) target point of view: Liu and Campillos [97] and Wassermann *et al.* [98] reported the comparison of 1640 ChemBank [99] assays and 150 HTS assays on the basis of their activity profiles, respectively. Both studies led to the discovery of biologically meaningful relationships between targets. Further, in-depth investigation of activity correlations across independent biochemical and cell-based assays could lead to a better understanding of similarities between proteins and could potentially further improve bioactivity modeling efforts (e.g. by expanding the applicability domain of proteochemometric modeling [100]). In pursuit of increased efficiency over conventional HTS campaigns, new screening paradigms have recently been suggested [3, 93]. These approaches increase (scaffold) hit rates at the expense of scaffold coverage, requiring balanced decision-making by the program team. Paricharak *et al.* [3] performed a large-scale validation of iterative screening based on Novartis HTS data. Herein, biological and chemical similarity metrics were used in parallel to iteratively expand around hits from multiple rounds of screening, resulting in significantly improved efficiency. Overall, screening 1% of the entire screening collection led to the retrieval of 7500 hits and a cumulative active scaffold coverage of 40%, with efficiency gains realized across a wide range of assay biology [3]. Maciejewski *et al.* [93] suggested an experimental design strategy depending on assay throughput and objective (e.g. hit retrieval or exploration of chemical space for model building). For systems allowing high throughput, conventional expansion around hits was suggested. By contrast, an active learning approach was considered best for iterative screening using smaller compound sets with the explicit aim of developing a model for later use. Here, active learning was preferred because of better sampling of chemical space. Finally, when the objective was to optimize cumulative (scaffold) hit rates in iterative screening, the ‘weak reinforcement strategy’ was suggested, where expansion around hits and exploration in under-sampled areas of chemical space were performed simultaneously [93].

## Conclusions

Although HTS has greatly gained momentum over the past decades, much profit can be realized by using intelligent measures to improve efficiency at the library design, hit triage and activity modeling stages. Data-driven approaches have consistently been used for improving these aspects, with the aim of systematically prioritizing structurally diverse sets of compounds for further interrogation. HTS-FP and the concept of bioactivity-based similarity have formed the basis for numerous studies showing remarkable improvements in hit retrieval and mode-of-action analyses. Moreover, analyses of activity correlations across independent biochemical and cell-based assays have resulted in promising preliminary discoveries of biologically meaningful relationships between targets. We believe that further investigation could lead to more unmapped insights into similarities between proteins and potentially improve bioactivity modeling efforts.

**Key Points**

- Consistently low hit rates and high upfront costs have prompted efforts to improve various aspects of HTS using heuristic measures, ranging from intelligent compound library design, through effective hit triage to bioactivity modeling to prioritize compounds for testing.
- Rapid progress in the area of bioactivity modeling has been made since the advent of the HTS fingerprint, a method of comparing compounds solely based on their bioactivity instead of chemical structure. Many studies showed significantly improved hit rates and mode-of-action analyses in screening campaigns.
- Recently, a public version of the HTS fingerprint based on PubChem data was released, which could be a promising resource for significantly improving activity modeling efforts in academic drug discovery.

## Notes

**Shardul Paricharak** is a PhD candidate in Cheminformatics at the Division of Medicinal Chemistry, Leiden Academic Centre for Drug Research, Leiden University. His research focuses on the development and application of *in silico* methods for improving bioactivity modeling.

## Notes

**Oscar Méndez-Lucio** is a postdoctoral researcher at the Pharmacy Department of the National Autonomous University of Mexico. His research focuses on the development of new computational methods for drug design.

## Notes

**Aakash Chavan Ravindranath** is currently a postdoctoral researcher at the Institute of Bioinformatics and Systems Biology of the Helmholtz Zentrum München, after obtaining his PhD from the University of Cambridge. His research focuses on understanding the mode of action of compounds that cause toxicity.

## Notes

**Andreas Bender** is a Reader for Molecular Informatics at the Department of Chemistry at the University of Cambridge. He is working on approaches of jointly analyzing chemical and biological data.

## Notes

**Ad IJzerman** is a professor of medicinal chemistry at the Leiden Academic Centre for Drug Research. He focuses on novel concepts in drug discovery, including allosteric modulation and target-binding kinetics.

## Notes

**Gerard van Westen** is an assistant professor in the research cluster Drug and Target Discovery at the Leiden Academic Centre for Drug Research. His main area of research is leveraging diverse and large-scale public data (‘big data’) with machine learning approaches for precision medicine.
